# Dose-response assessment of neuroactive botanical extracts and their bioactive constituents using microelectrode array (MEA) recordings in rat primary cortical cultures

**DOI:** 10.1080/13880209.2025.2583834

**Published:** 2025-11-07

**Authors:** Regina G. D. M. van Kleef, J. Pepijn Wopken, Julie Krzykwa, Constance A. Mitchell, Remco H. S. Westerink

**Affiliations:** a Neurotoxicology Research Group, Division of Toxicology, Institute for Risk Assessment Sciences (IRAS), Faculty of Veterinary Medicine, Utrecht University, Utrecht, The Netherlands; b Health and Environmental Sciences Institute, Washington, District of Columbia, USA

**Keywords:** Botanical safety testing, in vitro screening methods, micro-electrode array (MEA), neuronal activity, dose-response assessment

## Abstract

**Context:**

Use of botanicals is increasing. While often considered as safe, neuromodulatory effects have been demonstrated previously.

**Objective:**

Establish the neuroactive potential of 13 botanical extracts. For 8 of these, we made a direct comparison with their major constituent.

**Materials and methods:**

We used microelectrode array (MEA) recordings of primary rat cortical cultures to evaluate dose-response relationships on neuronal network function *in vitro*.

**Results:**

Exposure to extracts from milk thistle, usnea, green tea, aristolochia, and ephedra mildly inhibited neuronal activity with distinct neuronal activity phenotypes. Extracts of tripterygium, yohimbe, kratom, and kava strongly inhibited neuronal activity. Despite differences in the degree of inhibition, all inhibitory extracts show No Observed Effect Levels (NOELs) of 1–5 µg/mL. Exposure to aconite, blue cohosh, goldenseal and oleander induced specific activity phenotypes characterized by hyperexcitation and/or intensification of (network) burst activity. While the extracts exhibit a narrow effective dose range, the extracts from aconite, goldenseal and oleander are most potent with NOELs of 0.25–0.5 µg/mL.

Silybin B (milk thistle), epigallocatechin gallate (green tea), yohimbine (yohimbe), dihydrokavain (kava), mitragynine (kratom), aconitine (aconite), berberine (goldenseal) and oleandrin (oleander) were tested to investigate if the distinct neuronal activity phenotypes, indicative for the presence of multiple modes of action, are due to the known bioactive constituents of the extracts. For most constituents the activity phenotype is comparable to the extract, although the extract generally has a higher potency.

**Discussion and conclusion:**

Botanical extracts and constituents evoke diverse activity phenotypes, highlighting the complexity of hazard characterization of botanicals.

## Introduction

Botanicals have been widely used throughout history for a variety of purposes, including medicinal and dietary applications. Due to the growing interest in natural remedies and holistic approaches to health, the use of botanicals is widespread as an alternative for conventional pharmaceuticals, as natural cosmetic solutions, and as dietary supplements (Clarke et al. 2015; Smith et al. 2022). Consequently, botanical-based products like botanical-infused cosmetics, essential oils, and herbal supplements have gained widespread availability, use and consumption.

Despite this widespread use and the therapeutic benefits of many botanicals, it is essential to also consider possible adverse effects, such as potential botanical-drug interactions (e.g., St. John’s wort [*Hypericum perforatum* L., Hypericaceae]; Gurley et al. 2008). Reported adverse effects can in extreme cases be severe and result in permanent organ damage, including liver toxicity (e.g., comfrey [*Symphytum officinale* L., Boraginaceae] containing pyrrolizidine alkaloids; Brown et al. 2016), nephrotoxicity (e.g., *Aristolochia fangchi* Y.C. Wu ex L.D. Chou & S.M. Hwang and other *Aristolochia* spp., Aristolochiaceae containing aristolochic acids; Debelle et al. 2008), cardiotoxicity (e.g., ephedra [*Ephedra sinica* Stapf and other *Ephdera* spp., Ephedraceae]; National Toxicology Program 1986; Zell-Kanter et al. 2015), and neurotoxicity (e.g., aconite [*Aconitum napellus* L. and other *Aconitum* spp., Ranunculaceae] containing aconitine; Moritz et al. 2005). It is therefore imperative to establish safety testing methods. However, safety testing of botanicals can be challenging. On the one hand, this is due to variation in chemical composition and the concentration of bioactive compounds of botanicals which results from differences in growing conditions, extraction methods, and plant species used (Huie 2002; Mitchell et al. 2022). On the other hand, due to the chemical complexity and variability, botanicals can affect a multitude of cellular processes in a large number of organs, including the nervous system. Consequently, toxicity testing of botanicals requires either an extensive battery of assays to cover a large number of different modes of action or it should rely on overarching assays that functionally integrate the underlying processes (Kanungo et al. 2024).

For neurotoxicity testing, microelectrode array (MEA) recordings offer such an integrated approach. MEA recordings allow for assessment of changes in neuronal network function that result from underlying effects on for example ion channel and receptor function, neurotransmitter secretion and calcium homeostasis. MEA recordings are noninvasive, medium-throughput and show consistency across different laboratories (Vassallo et al. 2017). Primary rat cortical cultures, consisting mainly of glutamatergic and GABA-ergic neurons and supportive astrocytes (Hondebrink et al. 2016; Tukker et al. 2016), are the current gold standard for MEA recordings as these cultures rapidly and robustly develop spontaneous neuronal activity (Johnstone et al. 2010; Hogberg et al. 2011; McConnell et al. 2012; Gerber et al. 2021). Recently, we used MEA recordings to screen 16 different botanical extracts for their neuroactive characteristics. From those 16 botanical extracts, 13 showed clear effects on neuronal network activity, ranging from intense hyperexcitation to complete cessation of neuronal activity (van Kleef et al. 2024).

Our earlier data showed distinct activity patterns for different botanical extracts, suggesting different modes of action. Importantly, it was also shown that the activity patterns can be differentially affected by the exposure dose, with for example excitation at a low dose and inhibition of neuronal activity at a high dose. Moreover, at the lowest dose used for screening, several botanical extracts showed very profound effects, indicative of a high neuroactive potency. To advance hazard identification and potency ranking of botanicals upon acute exposure the aim of the present study is therefore to derive dose-response relationships for 13 neuroactive botanical extracts (Supplemental Table S1; see Mitchell et al. 2022; Kanungo et al. 2024 for details on the selection of botanicals). Additionally, we tested 8 known bioactive constituents in an attempt to better understand their contribution to the mixture effect and specific activity phenotypes (Supplemental Table S2). This work was conducted as a part of the Botanical Safety Consortium, which is made up of experts from government, industry, and academia working to develop screening strategies that can efficiently screen for botanical-induced toxicity, including neurotoxicity (Mitchell et al. 2022; Kanungo et al. 2024).

## Methods

### Chemicals and botanicals

All botanical test samples (Supplemental Table 1) were provided as dry extracts by the Botanical Safety Consortium (https://botanicalsafetyconsortium.org/; Mitchell et al. 2022), except for aconite [*Aconitum napellus* L., Ranunculaceae], oleander, [*Nerium oleander* L., Apocynaceae] and Tripterygium [*Trypterygium wilfordii* Hook. f., Celastraceae], which were provided as a stock solution of the extract (100, 88, and 55 mg/mL, respectively) in dimethyl sulfoxide (DMSO). Details on the sourcing, chemical analysis, and botanical details can be found at NIEHS’s CEBS website (https://cebs-ext.niehs.nih.gov/cebs/paper/15717), NIH’s database (https://doi.org/10.22427/NTP-DATA-500-007-001-000-3), in the supplemental materials, and is described in detail in Waidyanatha et al. (2024). Botanicals were selected based on existing literature with respect to toxicity or safety, from human (adverse event reporting and clinical trials), animal, or mechanistic studies, and because of their potential neurotoxicity (described in more detail in Kanungo et al. 2024) and demonstrated neuroactive potential (van Kleef et al. 2024).

To relate the effects of the tested extracts to individual constituents, we also tested eight botanical constituents (berberine, (-)-epigallocatechin-3-O-gallate, silybin B, yohimbine HCl, mitragynine, 7,8-dihydrokavain, aconitine, and oleandrin; Supplemental Table S2), which were provided by U.S. Pharmacopeia (USP), MRIGlobal and the National Institute of Environmental Health Sciences (NIEHS).

Phenol-red free neurobasal-A (NB-A) medium, l-glutamine (200 mM), penicillin/streptomycin (5000 U/mL/5000 mg/mL) and B-27 plus supplement were purchased from Life Technologies (Bleiswijk, the Netherlands). Unless otherwise noted, all other chemicals were obtained from Sigma-Aldrich (Zwijndrecht, the Netherlands). All solutions used in experiments, including control experiments, contained 0.1% DMSO.

### Cell culture

All animal experiments were performed in agreement with Dutch law, the European Community directives regulating animal research (2010/63/EU) and approved by the Ethical Committee for Animal Experiments of Utrecht University and the central committee for animal experimentation (AVD10800202115336). All efforts were made to minimize the number of animals used and their suffering.

Primary cultures of rat cortical neurons were prepared from pups born of timed-pregnant Wistar rats (Envigo, Horst, the Netherlands) on postnatal day 0 or 1 as described previously (Gerber et al. 2021). Briefly, rat pups were decapitated and the cortex was isolated and placed in ice-cold dissection medium (450 mL NBA medium, 14 g sucrose, 1.25 mL l-glutamine (200 mM), 5 mL glutamate (3.5 mM), 5 mL penicillin/streptomycin and 10 mL B-27 Plus Supplement, pH 7.4). Cortices were minced and triturated to a homogenous suspension and filtered through an easy strainer (100 μm, Greiner Bio One, Alphen aan den Rijn, The Netherlands). Subsequently, cells were centrifuged for 5 min at 800 rpm. The supernatant was removed and the pellet was resuspended using 1 mL of dissection medium per rat brain and diluted to a cell suspension containing 2 × 10^6^ cells/mL. Next, drops (50 µL/well) of cell-suspension were seeded on PEI (0,1% PEI solution in borate buffer (24 mM sodium borate/50 mM boric acid in Milli-Q adjusted to pH 8.4)) coated 48-well microelectrode array (MEA) plates (Axion Biosystems Inc., Atlanta, GA, USA) at a density of 1 x 10^5^ cells/well. Cells were allowed to attach in a humidified 5% CO_2_/95% air atmosphere for 2 h at 37 °C, before 450 µL dissection medium was added to each well. At four days *in vitro* (DIV 4), 450 µL dissection medium was replaced by 450 µL glutamate-free medium (450 mL NBA medium, 14 g sucrose, 1.25 mL l-glutamine (200 mM), 5 mL penicillin/streptomycin and 10 mL B-27 plus, pH 7.4). Cells were cultured in 5% CO_2_/95% air atmosphere at 37 °C until use at DIV 15. Additional details on all materials and experimental procedures are provided in Gerber et al. 2021, to enable full replication of the methods.

### MEA recordings

Multi-well MEA plates were used to record spontaneous neuronal activity. MEA plates contain 48 wells per plate, with per well an electrode array of 4 × 4 individual embedded microelectrodes (40–50 µm diameter; 350 µm center-to-center spacing), yielding a total of 768 electrodes, which can be used to record neuronal activity. Recordings were made as previously described (Gerber et al. 2021). All botanical extracts were tested at a dose of 50 µg/mL (limited by maximum solubility) down to the no-observed effect level (NOEL) to allow for a potency ranking. Each well was exposed to only one condition (i.e. no repeated dosing, but each well received only one dose of a botanical extract) to prevent potential effects of cumulative dosing such as (de)sensitization of neurotransmitter receptors and/or ion channels.

On DIV 15, a 48-well MEA plate was placed in a Maestro 768-channel amplifier with integrated heating system, temperature and CO_2_ controller, and data acquisition interface (Axion Biosystems Inc., Atlanta, GA, USA). Prior to each recording, MEA plates were allowed to equilibrate for around 5 min, after which a 30-minute baseline recording of spontaneous activity was started. Wells with at least four bursting electrodes and with a minimum of one network burst per minute at baseline recording were included for experiments. After the baseline recording, all wells were exposed individually by manually pipetting 55 µL of different doses of the extracts or vehicle (DMSO control) to each active well. Immediately after exposure, acute effects of botanical extracts on spontaneous neuronal activity (spiking and bursting behavior) were measured during a 30-minute recording at 37 °C. Additional details on all materials, software settings, and experimental procedures are provided in Gerber et al. 2021, to enable full replication of the methods.

### Data analysis and statistics

MEA data analysis was done as described in detail previously (Gerber et al. 2021). Briefly, MEA data acquisition was managed with Axion’s Integrated Studio (AxIS version 2.6). Raw data files were obtained by sampling channels simultaneously with a gain of 1000x and a sampling frequency of 12.5 kHz/channel using a band-pass filter (200–3000 Hz).

These raw data were pre-processed to obtain .spk files. Spikes were detected using the AxIS spike detector (Adaptive threshold crossing, Ada BandFlt v2) with a post/pre spike duration of 3.6/2.4 ms and a spike threshold of 7 × SD of the internal noise level (rms) of each individual electrode. Spike information was then further analyzed using NeuralMetrics Tool (v 3.1.7, Axion BioSystems) and custom-made macros in Excel. Bursts were defined using the Poisson surprise method (Legendy and Salcman 1985) with a minimum of 10 surprises. Network bursts were defined using an adaptive threshold with a minimum of 40 spikes, each separated by a maximum interval set automatically on a well-by-well basis based on the mean spike rate of each well, for a minimum of 15% of the electrodes/well. As we did not observe transient effects, data from the last 20 min of the 30-minute exposure recording were used for analysis, since this is the most stable timeframe for stable exposure effects (see Hondebrink et al. 2016).

Many different activity parameters can be derived from MEA recordings. For clarity, we focus on a selection of 8 parameters that have previously proven most important for assessing changes in neuronal activity. These include number of spikes, number of bursts, burst duration, number of spikes per burst, number of network bursts, network burst duration, number of spikes per network burst, and area under cross correlation as a measure for synchronicity (see Table S3 for further clarification of the different parameters).

Raster plots exemplifying the change in neuronal activity compared to baseline after exposure to the different extracts have been published previously (van Kleef et al. 2024). The acute effects of botanical extracts are presented as percentage change ± standard error of the mean (SEM) compared to baseline (treatment ratio), normalized to solvent control (DMSO, set at 100%). For each experimental condition, data represent average values of primary cultures derived from 9 to 37 wells (*n*) and 2–6 plates (N) from 2 to 4 independent preparations (except for some conditions that were tested only in 7–8 wells from 1 plate because complete cessation of activity was observed). The adjusted boxplot method was used to identify outliers (<5% outliers), which were excluded from further analysis. Benchmark response (BMR) cutoffs were set at 25%, which is based on the average variation in pooled DMSO control experiments. Effects that are smaller than the BMR are considered to be of limited toxicological relevance, even if significantly different from control. Statistical analyses were performed using GraphPad Prism (GraphPad Software, LLC version 10.4.1). Data were tested for significant effects using one-way ANOVA with significance values adjusted for multiple tests with Dunnett’s post-hoc test. A p-value ≤ 0.05 was considered statistically significant. Additional details on software settings and experimental procedures are provided in Gerber et al. 2021, to enable full replication of the analysis.

## Results

### Inhibitory botanical extracts

**Figure 1.**
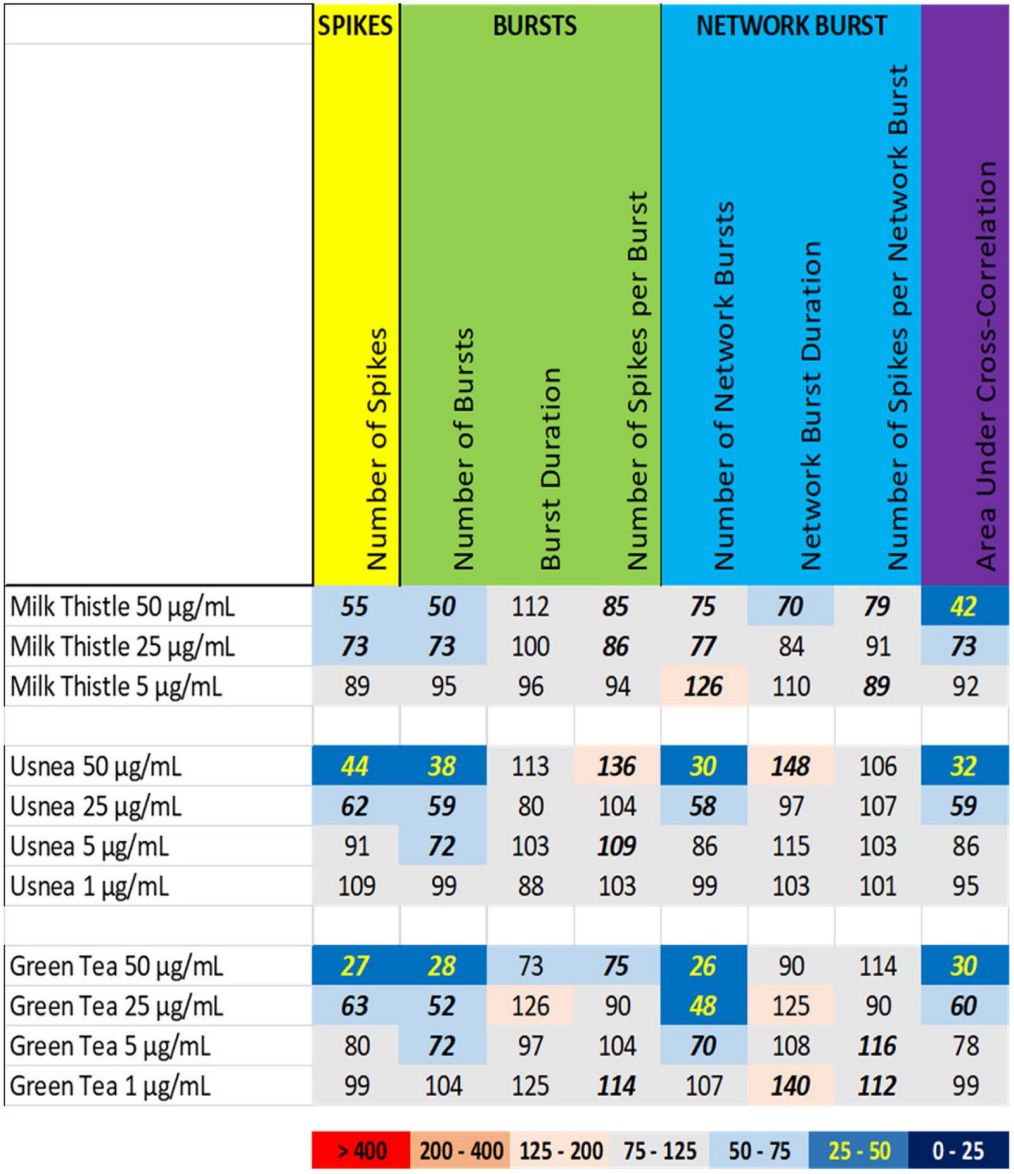
Overview of the effects of extracts of milk thistle (top), usnea (middle) and green tea (bottom) on eight neuronal activity parameters. Effects are expressed as a percentage of DMSO control and the degree of effect is indicated using a color scheme. Decreases are depicted in blue, increases are depicted in red. Values that do not exceed the benchmark response (BMR) of 25%, which is derived from the variation of DMSO controls, are depicted in light grey and are considered to be of limited toxicological relevance. Values in bold deviate significantly from DMSO control (*p* < 0.05).

Exposure to milk thistle, usnea, and green tea extracts resulted in a mild inhibition of neuronal activity of rat primary cortical cultures (Figure 1). Exposure to the extract of milk thistle (*Silybum marianum* (L.) Gaertn., Asteraceae) induced an inhibition of neuronal activity (Figure 1 (top), Supplemental Figure S1). Following exposure to milk thistle extract at 50 µg/mL, the number of spikes, the number of (network) bursts, and network burst duration were reduced. The area under cross correlation was also reduced, indicating a reduction in synchronicity. The inhibitory effect was attenuated following exposure at 25 µg/mL, whereas at 5 µg/mL the inhibitory effects were no longer observed.

Exposure to the usnea (*Usnea* spp., Parmeliaceae) extract at 50 µg/mL and 25 µg/mL resulted in a reduction in the number of spikes, the number of (network) bursts and the area under cross correlation (Figure 1 (middle), Supplemental Figure S2). Additionally, a mild increase in the number of spikes per burst, and the network burst duration was observed at 50 µg/mL. At 5 µg/mL, only the number of bursts was mildly reduced, and at 1 µg/mL usnea extract did not affect any of the neuronal activity parameters to a degree that exceeded the BMR.

Comparable to milk thistle and usnea, exposure to the extract of green tea (*Camellia sinensis* (L.) Kuntz, Theaceae) induced an inhibition of neuronal activity (Figure 1 (bottom), Supplemental Figure S3). At the high dose of 50 µg/mL, green tea extract exposure induced a profound reduction in the number of spikes, (network) bursts, and the area under cross correlation. Moreover, the burst duration and the number of spikes per bursts were decreased. At 25 µg/mL, the inhibitory effects on the number of spikes, (network) bursts and area under cross correlation were less pronounced, and at 5 µg/mL only a minor decrease in the number of (network) bursts was observed. Except for a mild increase in the network burst duration, at 1 µg/mL green tea extract did not affect any of the neuronal activity parameters to a degree that exceeds the BMR.

**Figure 2.**
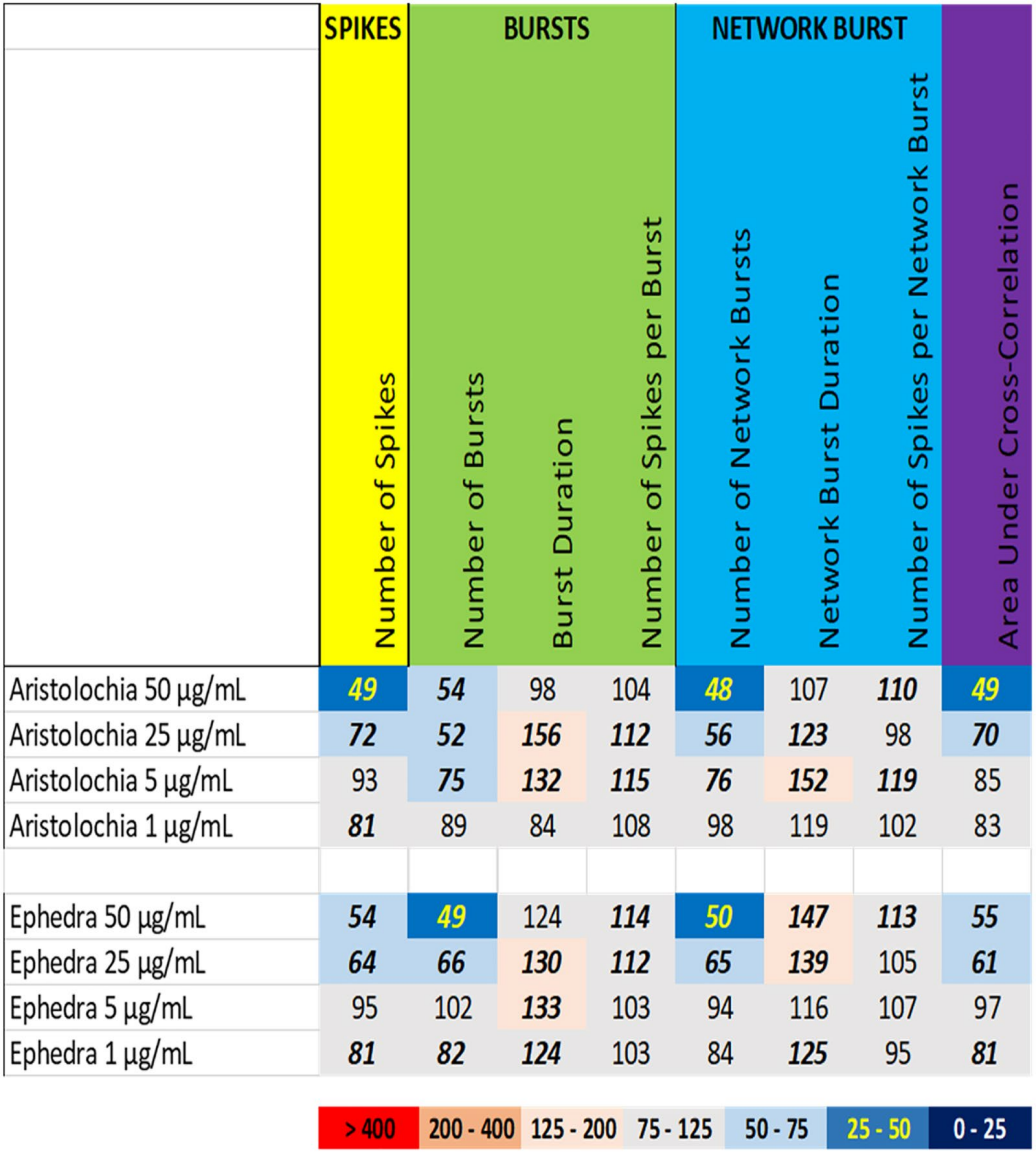
Overview of the effects of extracts of aristolochia (top) and ephedra (bottom) on eight neuronal activity parameters. Effects are expressed as a percentage of DMSO control and the degree of effect is indicated using a color scheme. Decreases are depicted in blue, increases are depicted in red. Values that do not exceed the benchmark response (BMR) of 25%, which is derived from the variation of DMSO controls, are depicted in light grey and are considered to be of limited toxicological relevance. Values in bold deviate significantly from DMSO control (*p* < 0.05).

Exposure to the extracts of aristolochia (*Aristolochia fangchi* Y.C. Wu ex L.D. Chou & S.M. Hwang, Aristolochiaceae) and ephedra (*Ephedra sinica* Stapf, Ephedraceae) resulted in a different activity phenotype with decreased overall activity and synchrony but increased (network) burst duration (Figure 2). At 50 and 25 µg/mL, aristolochia extract reduced the number spikes, the number of (network) bursts, and the area under cross correlation, which was paralleled by an increase in burst duration at 25 µg/mL. At 5 µg/mL only a modest decrease in the number of bursts and increase in the (network) burst duration was observed. At 1 µg/mL, aristolochia extract did not affect any of the neuronal activity parameters to a degree that exceeds the BMR (Figure 2 (top), Supplemental Figure S4).

Comparable to aristolochia, exposure to the extract of ephedra at 50 µg/mL and 25 µg/mL induced a decrease in the number of spikes, the number of (network) bursts and the area under cross correlation, which was paralleled by a minor increase in burst duration (25 and 5 µg/mL) and network burst duration (50 and 25 µg/mL). At 1 µg/mL, ephedra extract did not affect any of the neuronal activity parameters to a degree that exceeds the BMR (Figure 2 (bottom), Supplemental Figure S5).

**Figure 3.**
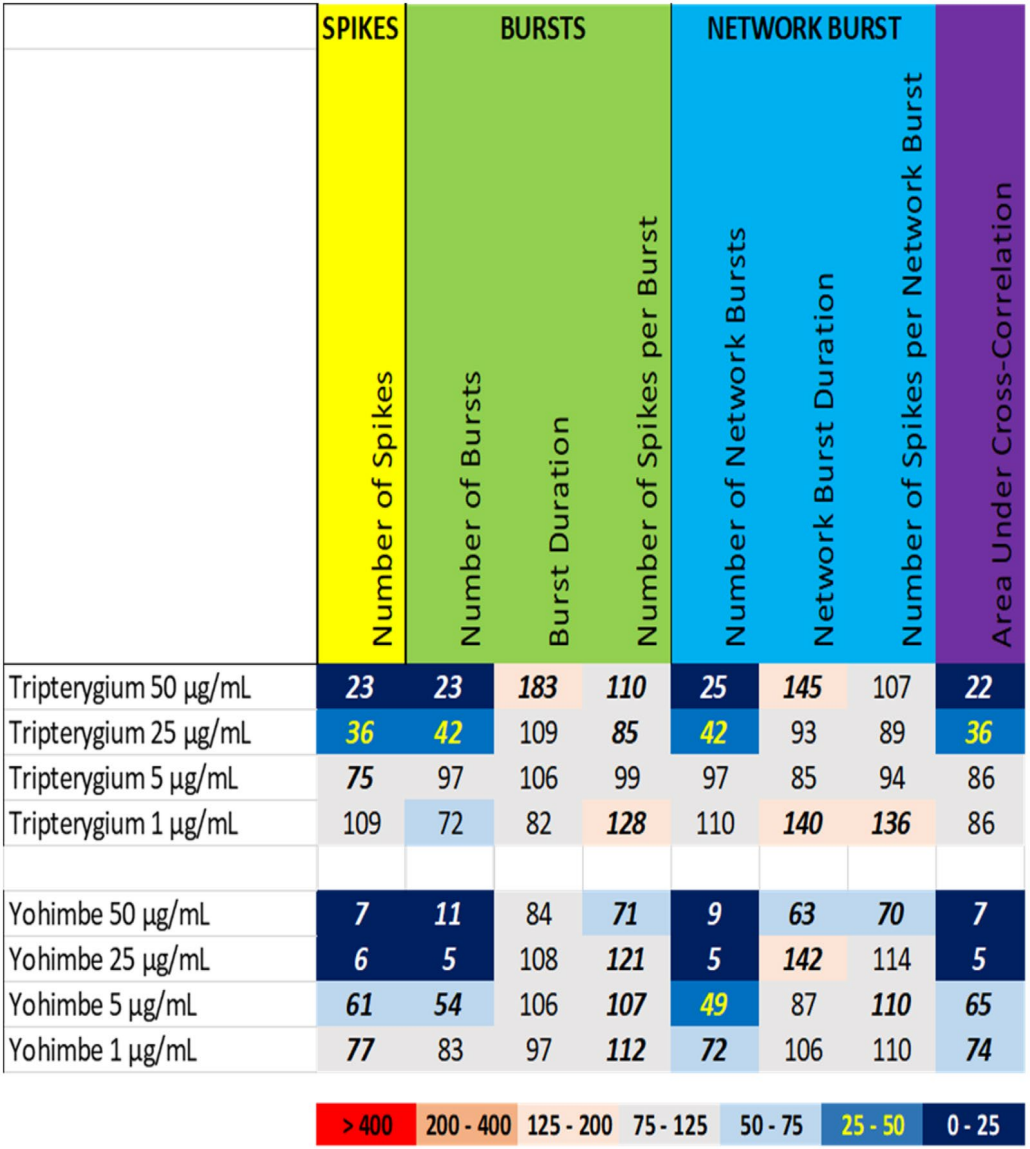
Overview of the effects of extracts of tripterygium (top) and yohimbe (bottom) on eight neuronal activity parameters. Effects are expressed as a percentage of DMSO control and the degree of effect is indicated using a color scheme. Decreases are depicted in blue, increases are depicted in red. Values that do not exceed the benchmark response (BMR) of 25%, which is derived from the variation of DMSO controls, are depicted in light grey and are considered to be of limited toxicological relevance. Values in bold deviate significantly from DMSO control (*p* < 0.05).

Exposure to the extracts of tripterygium [*Trypterygium wilfordii* Hook. f., Celastraceae] and yohimbe (*Corynanthe johimbe*, syn. *Pausinystalia johimbe* (K. Schum.) Pierre ex Beille, Rubiaceae) induced a potent inhibition of neuronal activity (Figure 3). At 50 µg/mL and 25 µg/mL, tripterygium extract reduced the number spikes, the number of (network) bursts, and the area under cross correlation, which was paralleled by a modest increase in (network) burst duration at 50 µg/mL. At 5 µg/mL and 1 µg/mL, tripterygium extract evoked no or only minor changes in neuronal activity parameters (Figure 3 (top), Supplemental Figure S6). At 50 μg/mL, the extract of triperygium induced a modest decrease in mitochondrial activity (25%; van Kleef et al. 2024), suggesting that the observed inhibition of neuronal activity at this dose could be partly due to a decrease in cell viability.

Exposure to the extract of yohimbe (*Corynanthe johimbe*, syn. *Pausinystalia johimbe* (K. Schum.) Pierre ex Beille, Rubiaceae) at 50 µg/mL and 25 µg/mL resulted in a profound reduction in the number of spikes, the number of (network) bursts and the area under cross correlation. At 50 µg/mL, a modest reduction in the network burst duration and the number of spikes per (network) burst was observed, although at 25 µg/mL, the network burst duration was even slightly increased. At 5 µg/mL, the reduction in the number of spikes, the number of (network) bursts and the area under cross correlation was less pronounced and at 1 µg/mL, yohimbe extract induced only a mild reduction in the number of network bursts and the area under cross correlation (Figure 3 (bottom), Supplemental Figure S7). At 50 μg/mL, the extract of yohimbe induced a decrease in mitochondrial activity (39%; van Kleef et al. 2024), suggesting that the observed inhibition of neuronal activity at this dose could be partly due to a decrease in cell viability.

**Figure 4.**
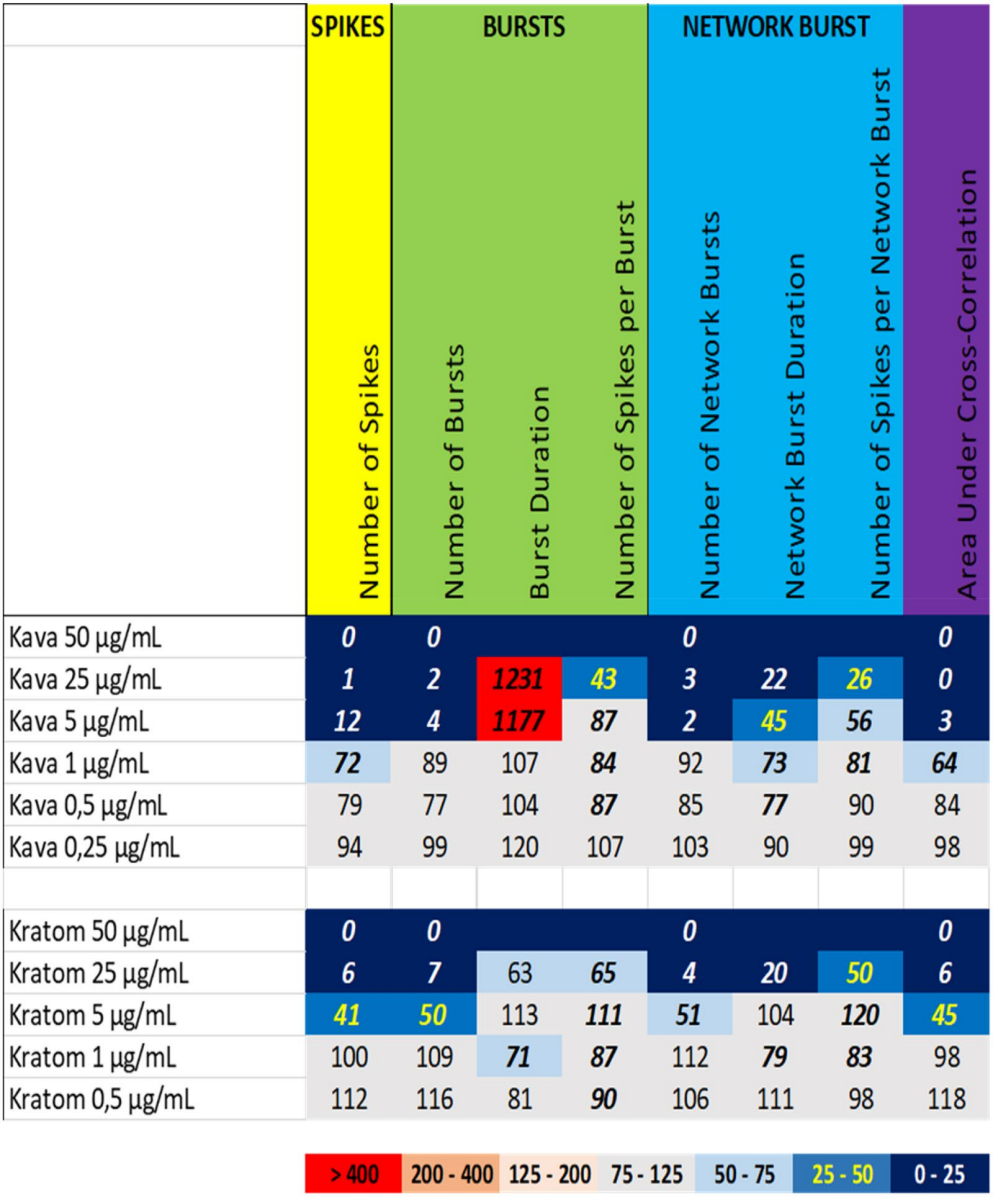
Overview of the effects of extracts of kava (top) and kratom (bottom) on eight neuronal activity parameters. Effects are expressed as a percentage of DMSO control and the degree of effect is indicated using a color scheme. Decreases are depicted in blue, increases are depicted in red. Values that do not exceed the benchmark response (BMR) of 25%, which is derived from the variation of DMSO controls, are depicted in light grey and are considered to be of limited toxicological relevance. Values in bold deviate significantly from DMSO control (*p* < 0.05).

Exposure to kava (*Piper methysticum* G. Forst., Piperaceae) and kratom (*Mitragyna speciosa* (Korth.) Havil., Rubiaceae) extracts resulted in a potent inhibition of neuronal activity (Figure 4). Exposure of cortical cultures to kava extract at 50 µg/mL completely abolished neuronal activity. At 25 µg/mL and 5 µg/mL, all neuronal activity parameters were strongly reduced, except burst duration, which showed a remarkable increase. At 1 µg/mL, the number spikes, network burst duration, and the area under cross correlation were still reduced, but at 0.5 µg/mL and 0.25 µg/mL, Kava extract did not affect any of the neuronal activity parameters to a degree that exceeds the BMR (Figure 4 (top), Supplemental Figure S8).

Comparable to kava, exposure to the extract of kratom at 50 µg/mL resulted in the complete cessation of neuronal activity (Figure 4 (bottom), Supplemental Figure S9). At 25 µg/mL, the kratom extract strongly decreased all neuronal parameters, whereas at 5 µg/mL only the number of spikes, the number of (network) bursts and the area under cross correlation were significantly decreased to a degree that exceeds the BMR. At 1 µg/mL and 0.5 µg/mL, kratom extract evoked no or only minor changes in neuronal activity parameters.

### Botanical extracts with a specific or excitatory neuronal activity phenotype

**Figure 5.**
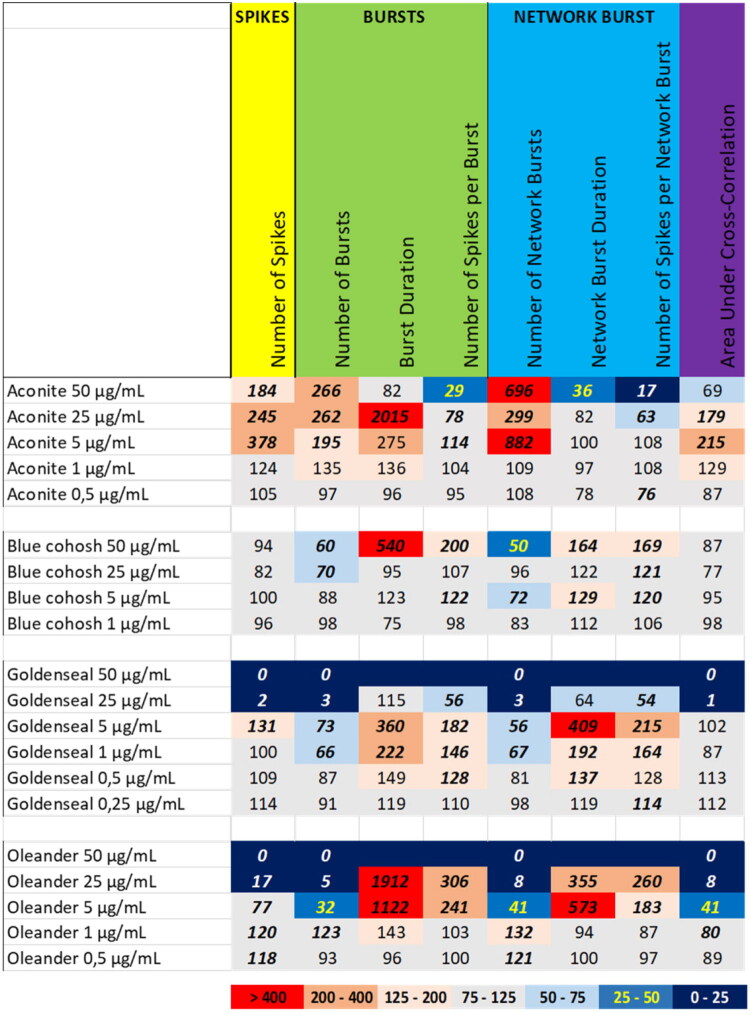
Overview of the effects of extracts of aconite (top), blue cohosh (upper Middle), goldenseal (lower Middle) and oleander (bottom) on eight neuronal activity parameters. Effects are expressed as a percentage of DMSO control and the degree of effect is indicated using a color scheme. Decreases are depicted in blue, increases are depicted in red. Values that do not exceed the benchmark response (BMR) of 25%, which is derived from the variation of DMSO controls, are depicted in light grey and are considered to be of limited toxicological relevance. Values in bold deviate significantly from DMSO control (*p* < 0.05).

As described above, most selected botanical extracts inhibit neuronal activity. However, the extracts from aconite, blue cohosh (*Caulophyllum thalictroides* (L.) Michx., Berberidaceae), goldenseal (*Hydrastis canadensis* L., Ranunculaceae), and oleander (*Nerium oleander* L., Apocynaceae) induce specific and/or excitatory neuronal activity phenotypes (Figure 5). Exposure to the extract of aconite at 50 µg/mL increased the number of spikes and (network) burst, but reduced the number of spikes per (network) burst as well as the network burst duration and the area under cross correlation. At 25 µg/mL and 5 µg/mL, aconite extract not only increased the number of spikes and (network) burst, but also induced a profound increase in the burst duration and the area under cross correlation. At 1 µg/mL and 0.5 µg/mL, aconite extract evoked no or only minor changes in neuronal activity parameters (Figure 5 (top), Supplemental Figure S10).

Exposure to the extract of blue cohosh induced a specific neuronal activity phenotype with (network) burst intensification. At 50 µg/mL, blue cohosh extract reduced the number of (network) bursts, which was paralleled by a remarkable increase in (network) burst duration and in the number of spikes per (network) burst. At 25 µg/mL and 5 µg/mL only a modest decrease in the number of (network) bursts could still be observed, but the intensified (network) bursts were no longer observed. At 1 µg/mL, blue cohosh extract did not affect any of the neuronal activity parameters to a degree that exceeds the BMR (Figure 5 (upper middle), Supplemental Figure S11).

Exposure to the extract of goldenseal induced a biphasic neuronal activity phenotype. At 50 µg/mL and 25 µg/mL, exposure to goldenseal extract resulted in the (near) complete cessation of neuronal activity. At the 5 µg/mL, however, the number of (network) bursts was reduced, whereas the number of spikes, (network) burst duration and the number of spikes per (network) burst were markedly increased. At 1 µg/mL, these effects were less pronounced and at 0.5 µg/mL, only a modest and/or non-significant increase in (network) burst duration and the number of spikes per (network) burst could be observed. At 0.25 µg/mL, goldenseal extract did not affect any of the neuronal activity parameters to a degree that exceeds the BMR (Figure 5 (lower middle), Supplemental Figure S12). At 50 μg/mL, the extract of goldenseal induced a mild decrease in mitochondrial activity (9%; van Kleef et al. 2024), suggesting that the observed inhibition of neuronal activity at this dose could be partly due to a decrease in cell viability.

Exposure to the extract of oleander at 50 µg/mL resulted in a complete cessation of neuronal activity. At 25 µg/mL, the extract of oleander strongly decreased the number of spikes and (network) bursts as well as the area under cross correlation, whereas the duration of the remaining (network) bursts and the number of spikes per (network) burst were strongly increased. At 5 µg/mL, the intensification of the (network) bursts was less pronounced and at 1 µg/mL, only a minor and/or non-significant increase in burst duration and the number of network bursts could be observed. At 0.5 µg/mL, oleander extract did not affect any of the neuronal activity parameters to a degree that exceeds the BMR (Figure 5 (bottom), Supplemental Figure S13).

### Comparison of botanical extracts with their constituents

In addition to the botanical extracts, we tested eight constituents that are known to induce bioactivity (silybin B, epigallocatechin gallate, yohimbine, dihydrokavain, mitragynine, aconitine, berberine and oleandrin; see Supplemental Table S2 for concentrations of constituents in the extracts and how the tested concentrations of constituents relate to the different tested dose levels of the extracts) in an attempt to better understand their contribution to the mixture effect and specific activity phenotypes.

**Figure 6.**
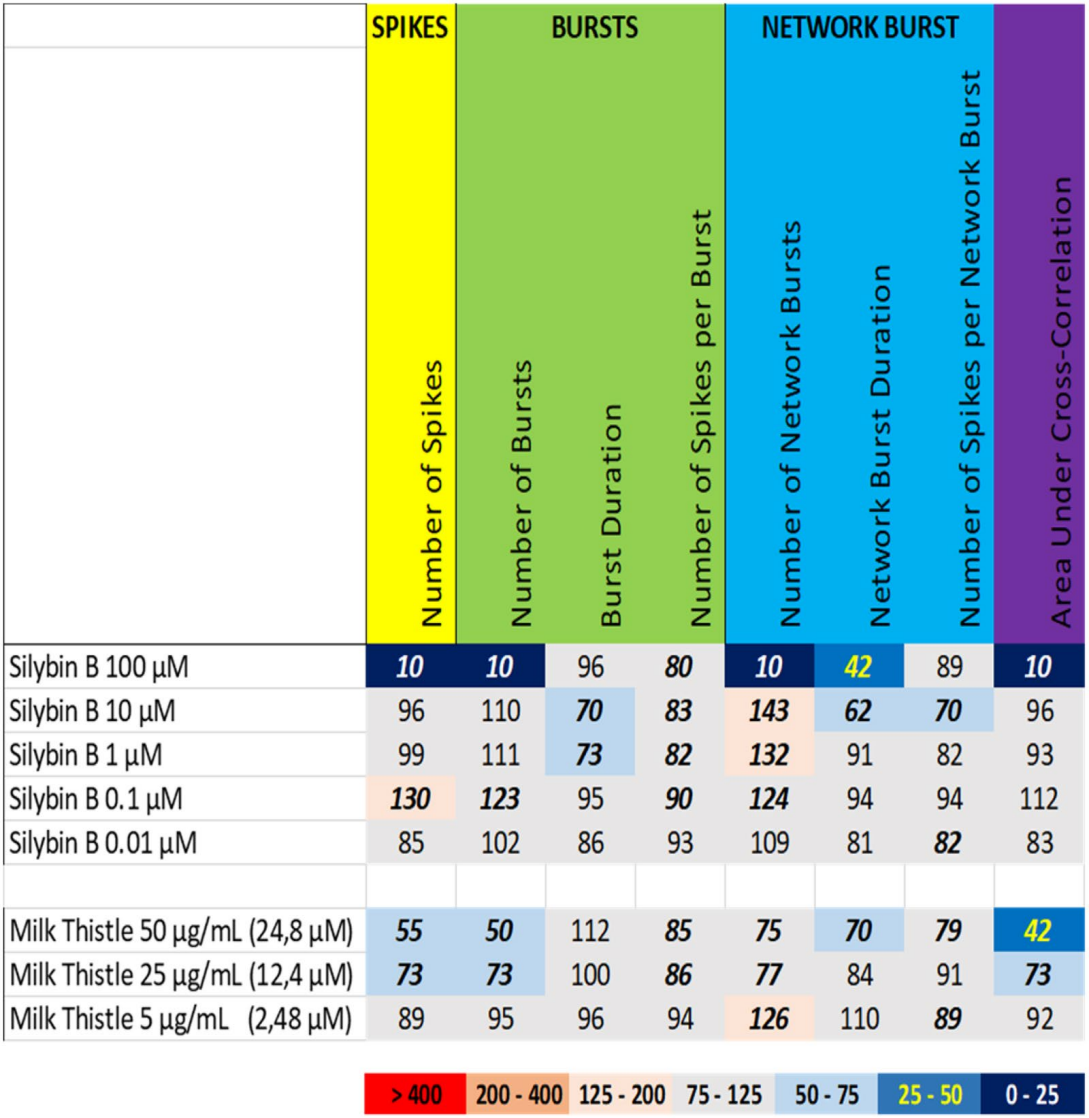
Overview of the effects of silybin B (top) compared to the extract of milk thistle (bottom) on eight neuronal activity parameters. Effects are expressed as a percentage of DMSO control and the degree of effect is indicated using a color scheme. Decreases are depicted in blue, increases are depicted in red. Values that do not exceed the benchmark response (BMR) of 25%, which is derived from the variation of DMSO controls, are depicted in light grey and are considered to be of limited toxicological relevance. Values in bold deviate significantly from DMSO control (*p* < 0.05). The concentration of silybin B in milk thistle extract is indicated in between parentheses.

Compared to milk thistle, exposure to the constituent silybin B resulted in a different activity phenotype. While milk thistle induced a mild inhibition of neuronal activity at 50 µg/mL and 25 µg/mL (equivalent to 25 µM and 12.4 µM silybin B, respectively) with a decreased number of spikes, number of (network) bursts and area under cross-correlation (Figure 1 (top), Supplemental Figure S1), silybin B at 100 µM induced a profound reduction in the number of spikes, the number of (network) bursts, and the area under cross correlation, which was accompanied by a modest reduction in network burst duration. Contrary, at 10 µM and 1 µM, silybin B mildly increased the number of network bursts, which was accompanied by a modest decrease in the (network) burst duration and the number of spikes per network burst (Figure 6 (top), Supplemental Figure S14). At 0.1 µM and 0.01 µM, silybin B evoked no or only minor changes in neuronal activity parameters.

**Figure 7.**
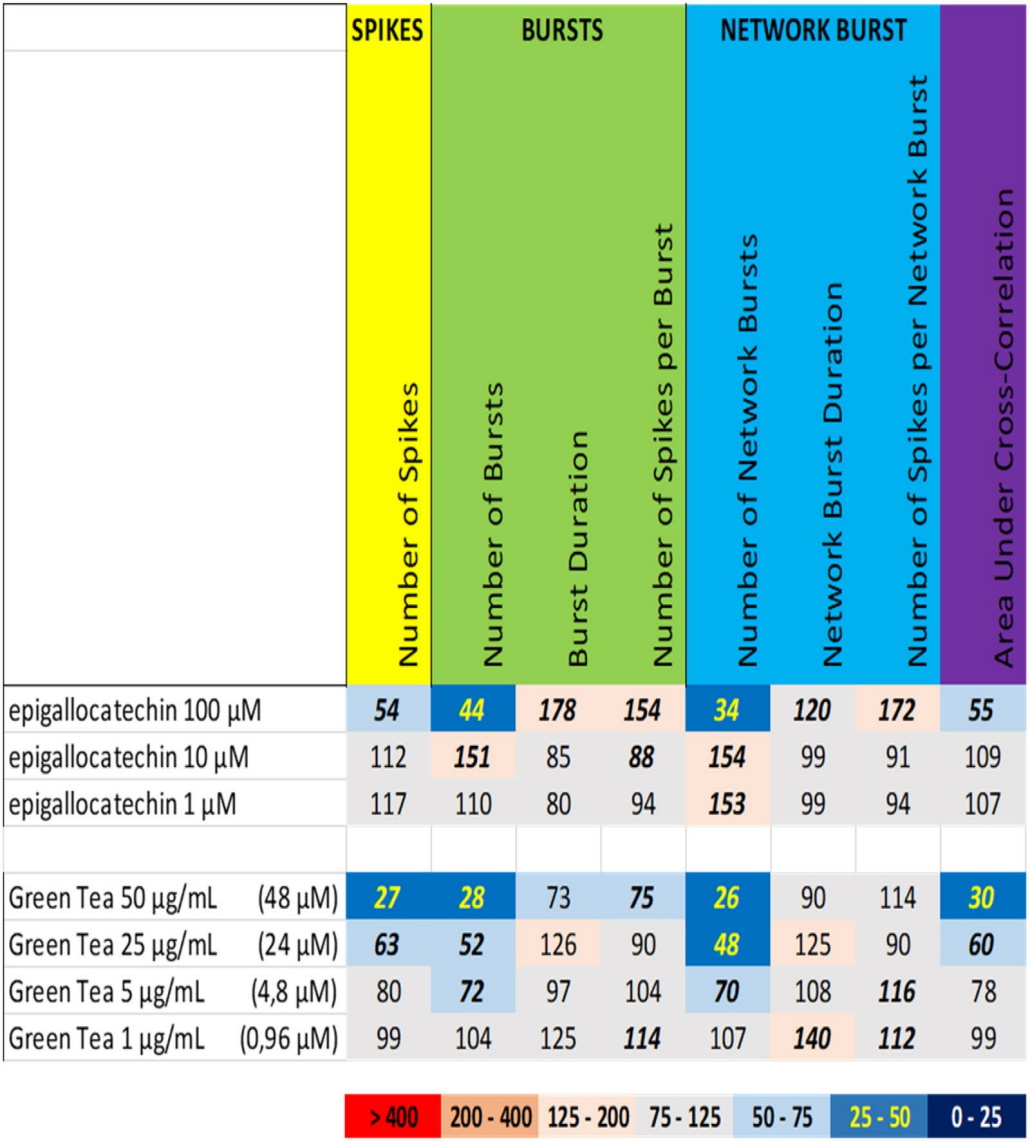
Overview of the effects of epigallocatechin gallate (top) compared to the extract of green tea (bottom) on eight neuronal activity parameters. Effects are expressed as a percentage of DMSO control and the degree of effect is indicated using a color scheme. Decreases are depicted in blue, increases are depicted in red. Values that do not exceed the benchmark response (BMR) of 25%, which is derived from the variation of DMSO controls, are depicted in light grey and are considered to be of limited toxicological relevance. Values in bold deviate significantly from DMSO control (*p* < 0.05). The concentration of epigallocatechin gallate in green tea extract is indicated in between parentheses.

**Figure 8.**
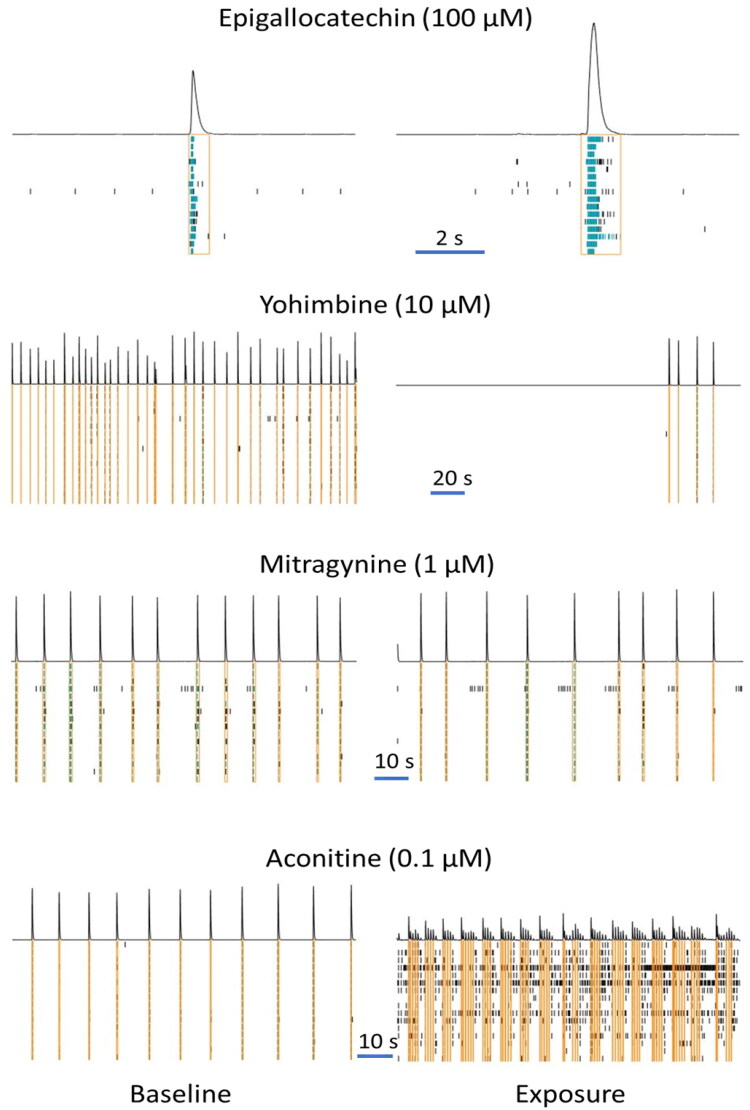
Raster plot showing the activity of the neuronal network in a single, representative well before (baseline, left) and after exposure (right) to 100 µm epigallocatechin gallate (top), 10 µM yohimbine (upper Middle), 1 µM mitragynine (lower Middle) and 0,1 µM aconitine (bottom). Activity is recorded at 16 different electrodes and is depicted in black (spikes) and cyan (bursts) ticks and in orange boxes (network burst). The trace on top of the recording shows the cumulative activity recorded at the 16 electrodes. Note the differences in time scale, indicated by the horizontal blue calibration line.

Green tea extract (Figure 7 (bottom), Supplemental Figure S3) at 50 µg/mL (equivalent to 48 µM epigallocatechin gallate), induced a profound reduction in the number of spikes, the number of (network) bursts, and the area under cross correlation, which was attenuated at 25 µg/mL (equivalent to 24 µM epigallocatechin gallate). However, exposure to epigallocatechin gallate at 100 µM resulted in a different activity phenotype, with inhibition of the number of spikes, the number of (network) bursts, and the area under cross correlation, although this was paralleled by a modest increase in the burst duration and the number of spikes per (network) burst (Figure 7 (top), Supplemental Figure S15; also see raster plot example in Figure 8 (top)). Contrary, epigallocatechin gallate at 10 µM mildly increased the number of (network) bursts. At 1 µM, epigallocatechin gallate evoked no or only minor changes in neuronal activity parameters. So, compared to the extracts of milk thistle and green tea, the constituents silybin-B and epigallocatechin gallate do not result in a comparable activity phenotype.

**Figure 9.**
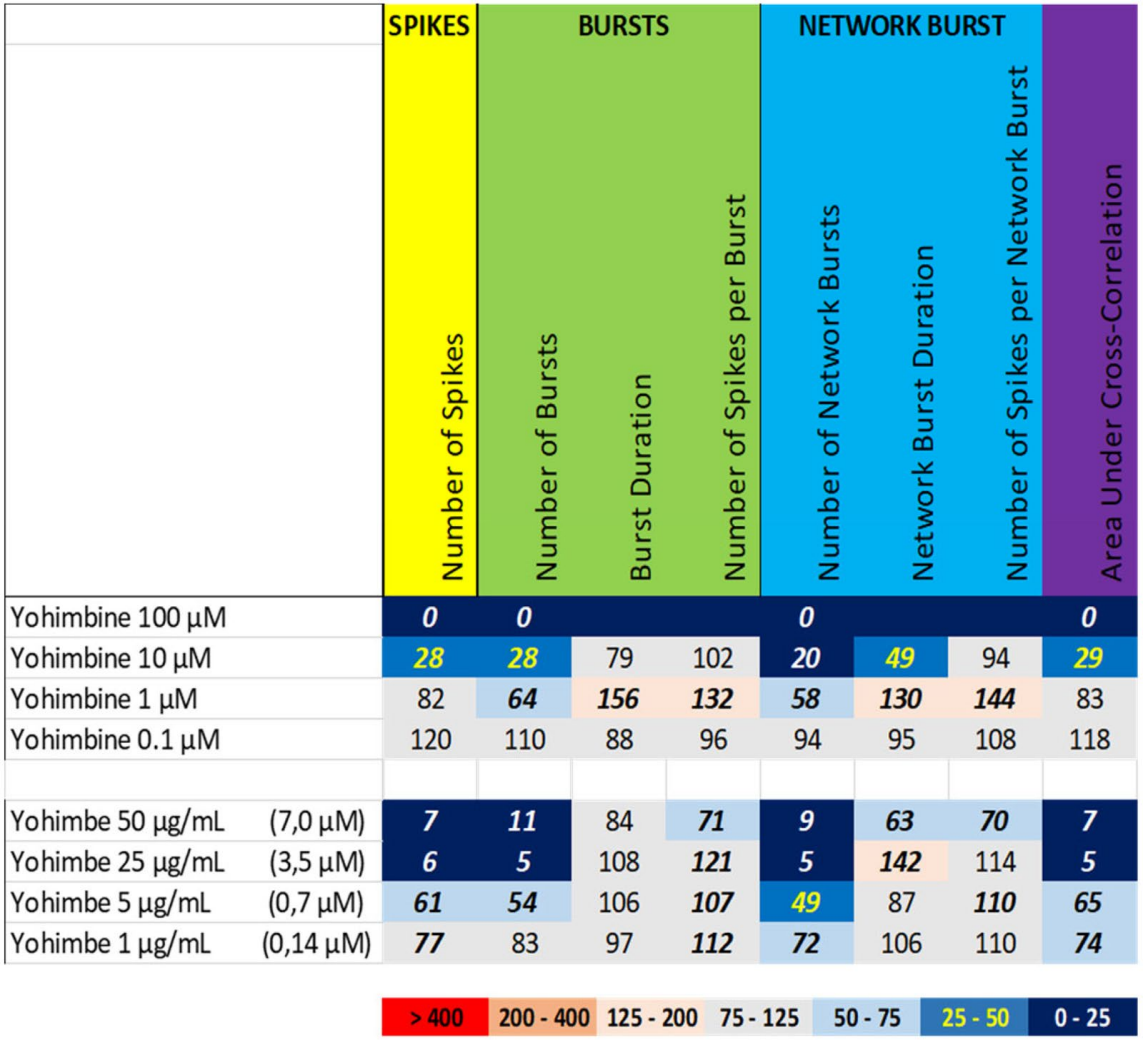
Overview of the effects of yohimbine (top) compared to the extract of yohimbe (bottom) on eight neuronal activity parameters. Effects are expressed as a percentage of DMSO control and the degree of effect is indicated using a color scheme. Decreases are depicted in blue, increases are depicted in red. Values that do not exceed the benchmark response (BMR) of 25%, which is derived from the variation of DMSO controls, are depicted in light grey and are considered to be of limited toxicological relevance. Values in bold deviate significantly from DMSO control (*p* < 0.05). The concentration of yohimbine in yohimbe extract is indicated in between parentheses.

Exposure to the extract of yohimbe at 25 µg/mL and 50 µg/mL (equivalent to 3.5 µM and 7 µM yohimbine, respectively) resulted in a profound reduction in the number of spikes, the number of (network) bursts, and the area under cross correlation (Figure 9 (bottom), Figure 8 (upper middle), Supplemental Figure S7). While yohimbine to a large extent resembled the inhibitory activity phenotype, with a decrease in the number of spikes, the number of (network) bursts, and the area under cross correlation (Figure 9 (top), Supplemental Figure S16), this reduction was only observed at concentrations ≥10 µM, highlighting the potency of the extract compared to yohimbine alone.

**Figure 10.**
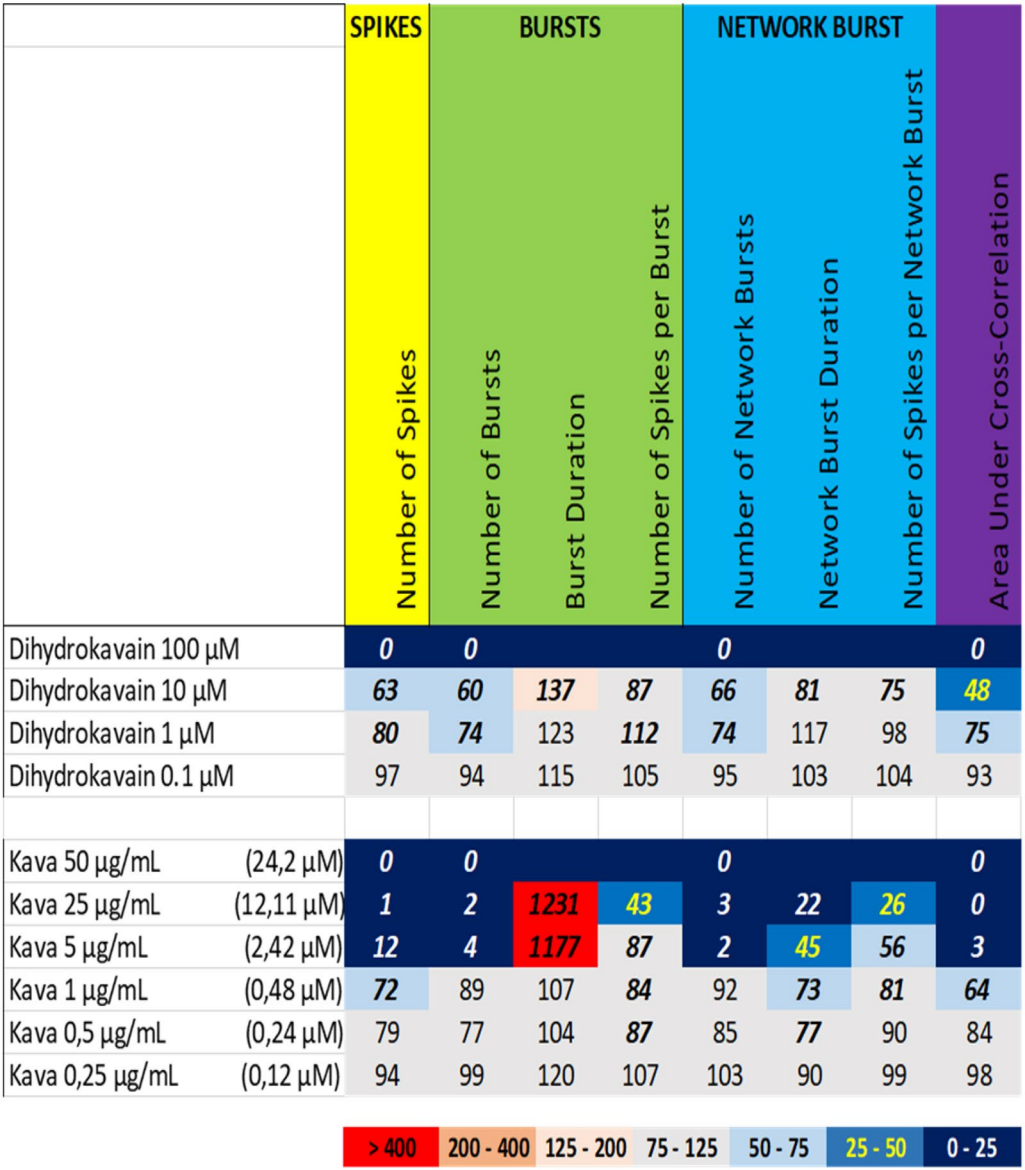
Overview of the effects of dihydrokavain (top) compared to the extract of kava (bottom) on eight neuronal activity parameters. Effects are expressed as a percentage of DMSO control and the degree of effect is indicated using a color scheme. Decreases are depicted in blue, increases are depicted in red. Values that do not exceed the benchmark response (BMR) of 25%, which is derived from the variation of DMSO controls, are depicted in light grey and are considered to be of limited toxicological relevance. Values in bold deviate significantly from DMSO control (*p* < 0.05). The concentration of dihydrokavain in kava extract is indicated in between parentheses.

Exposure to kava extract at 25 µg/mL and 50 µg/mL (equivalent to 12.1 µM and 24.2 µM dihydrokavain, respectively) resulted in a (near) complete inhibition of neuronal activity. Also at 5 µg/mL (equivalent to 2.4 µM dihydrokavain), all neuronal activity parameters were strongly reduced (Figure 10 (bottom), Supplemental Figure S8), except for burst duration which was strongly increased at 25 µg/mL and 5 µg/mL. While dihydrokavain at 100 µM also resulted in a complete cessation of neuronal activity, 10 µM resulted only in a mild reduction of the number of spikes and (network) bursts, whereas concentrations ≤ 1 µM were largely ineffective (Figure 10 (top), Supplemental Figure S17).

**Figure 11.**
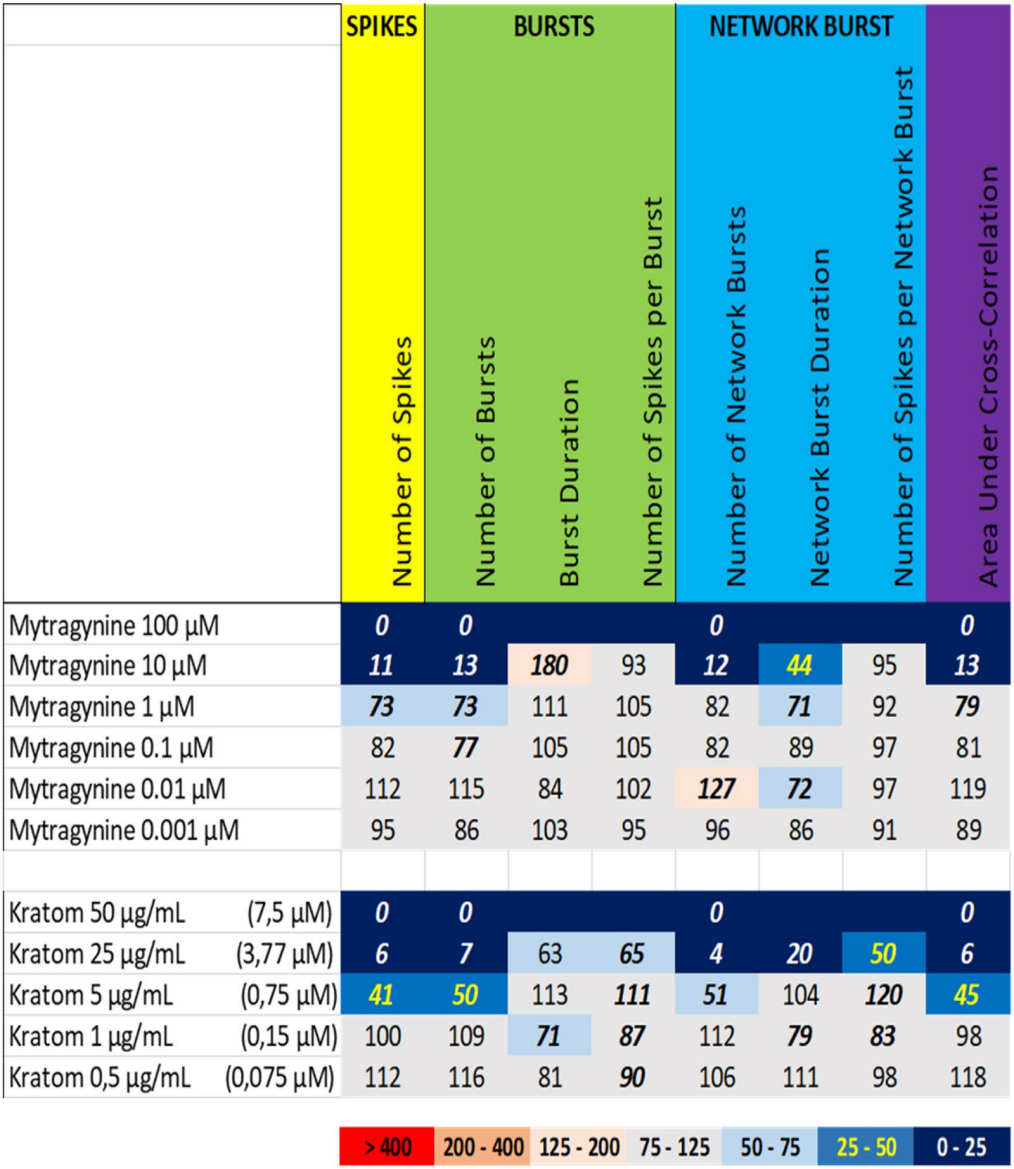
Overview of the effects of mitragynine (top) compared to the extract of kratom (bottom) on eight neuronal activity parameters. Effects are expressed as a percentage of DMSO control and the degree of effect is indicated using a color scheme. Decreases are depicted in blue, increases are depicted in red. Values that do not exceed the benchmark response (BMR) of 25%, which is derived from the variation of DMSO controls, are depicted in light grey and are considered to be of limited toxicological relevance. Values in bold deviate significantly from DMSO control (*p* < 0.05). The concentration of mytragynine in the kratom extract is indicated in between parentheses.

Exposure to kratom extract at 25 µg/mL and 50 µg/mL (equivalent to 3.8 µM and 7.5 µM mitragynine, respectively) resulted in a (near) complete inhibition of neuronal activity. Also at 5 µg/mL (equivalent to 0.75 µM mitragynine), the number of spikes, the number of (network) bursts and the area under cross correlation were reduced (Figure 11 (bottom), Supplemental Figure S9). Exposure to mitragynine at 10 µM and 100 µM also resulted in a (near) complete cessation of neuronal activity, whereas mitragynine at 1 µM resulted only in a mild reduction of the number of spikes, the number of bursts, and the area under cross correlation (Figure 11 (top), Figure 8 (lower middle), Supplemental Figure S18). While the activity phenotype of mitragynine thus resembles that of the kratom extract, the extract is more potent than mitragynine alone.

**Figure 12.**
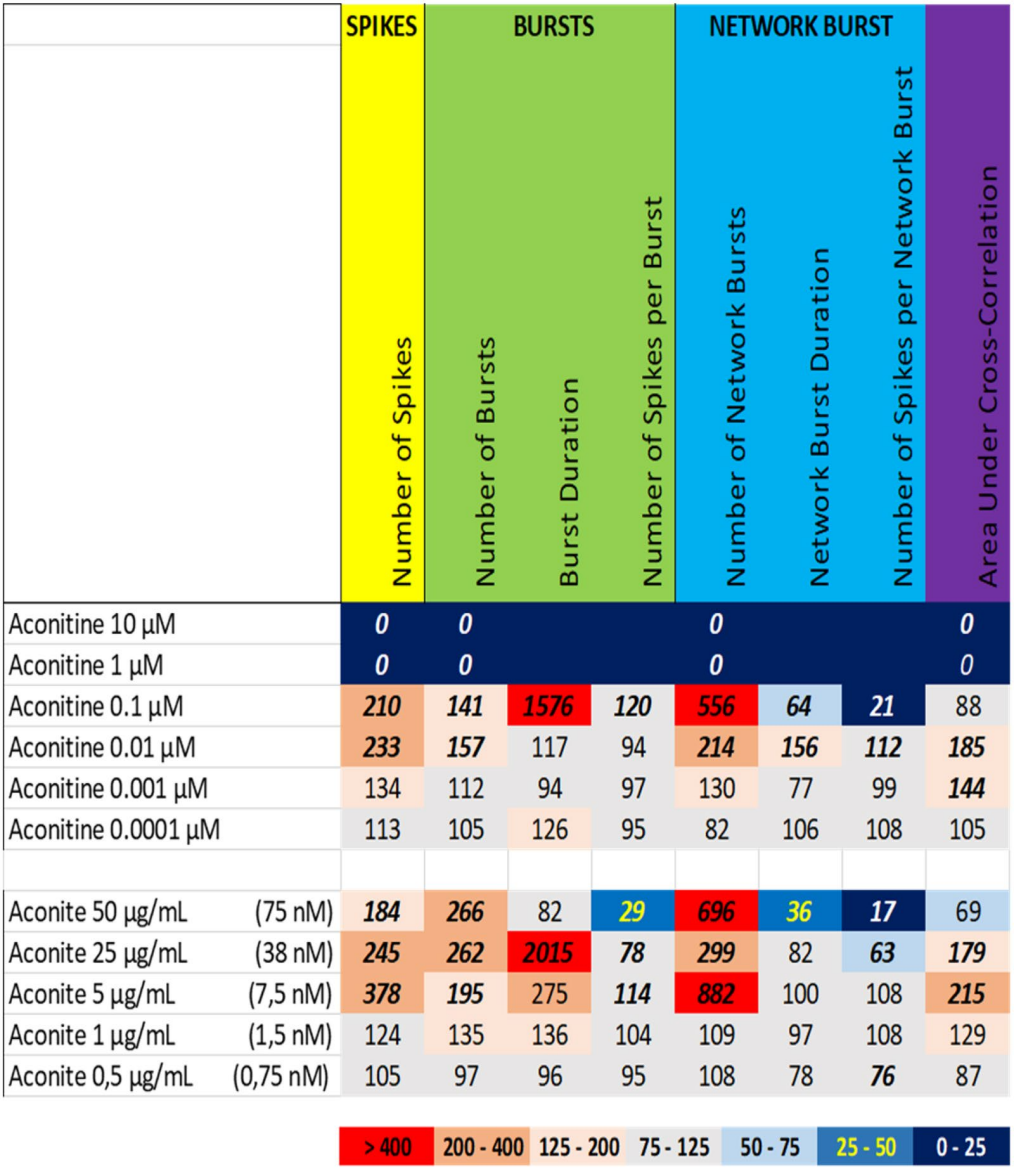
Overview of the effects of aconitine (top) compared to the extract of aconite (bottom) on eight neuronal activity parameters. Effects are expressed as a percentage of DMSO control and the degree of effect is indicated using a color scheme. Decreases are depicted in blue, increases are depicted in red. Values that do not exceed the benchmark response (BMR) of 25%, which is derived from the variation of DMSO controls, are depicted in light grey and are considered to be of limited toxicological relevance. Values in bold deviate significantly from DMSO control (*p* < 0.05). The concentration of aconitine in the aconite extract is indicated in between parentheses.

Exposure to the extract of aconite at 5 µg/mL, 25 µg/mL and 50 µg/mL (equivalent to 7.5 nM, 38 nM and 75 nM aconitine, respectively) resulted in an increase in the number of spikes and (network) burst, and the burst duration (Figure 12 (bottom), Supplemental Figure S10), which at the highest dose was accompanied by a clear reduction in the number of spikes per (network) burst and in network burst duration. Exposure to aconitine at 100 nM resulted in a comparable activity phenotype, although at 10 nM the excitatory effect is already strongly attenuated. The activity phenotype of aconitine thus resembles that of the aconite extract, although the extract is more potent than aconitine alone. Notably, high concentrations of aconitine induced a complete inhibition of neuronal activity (Figure 12 (top), Figure 8 (bottom), Supplemental Figure S19).

**Figure 13.**
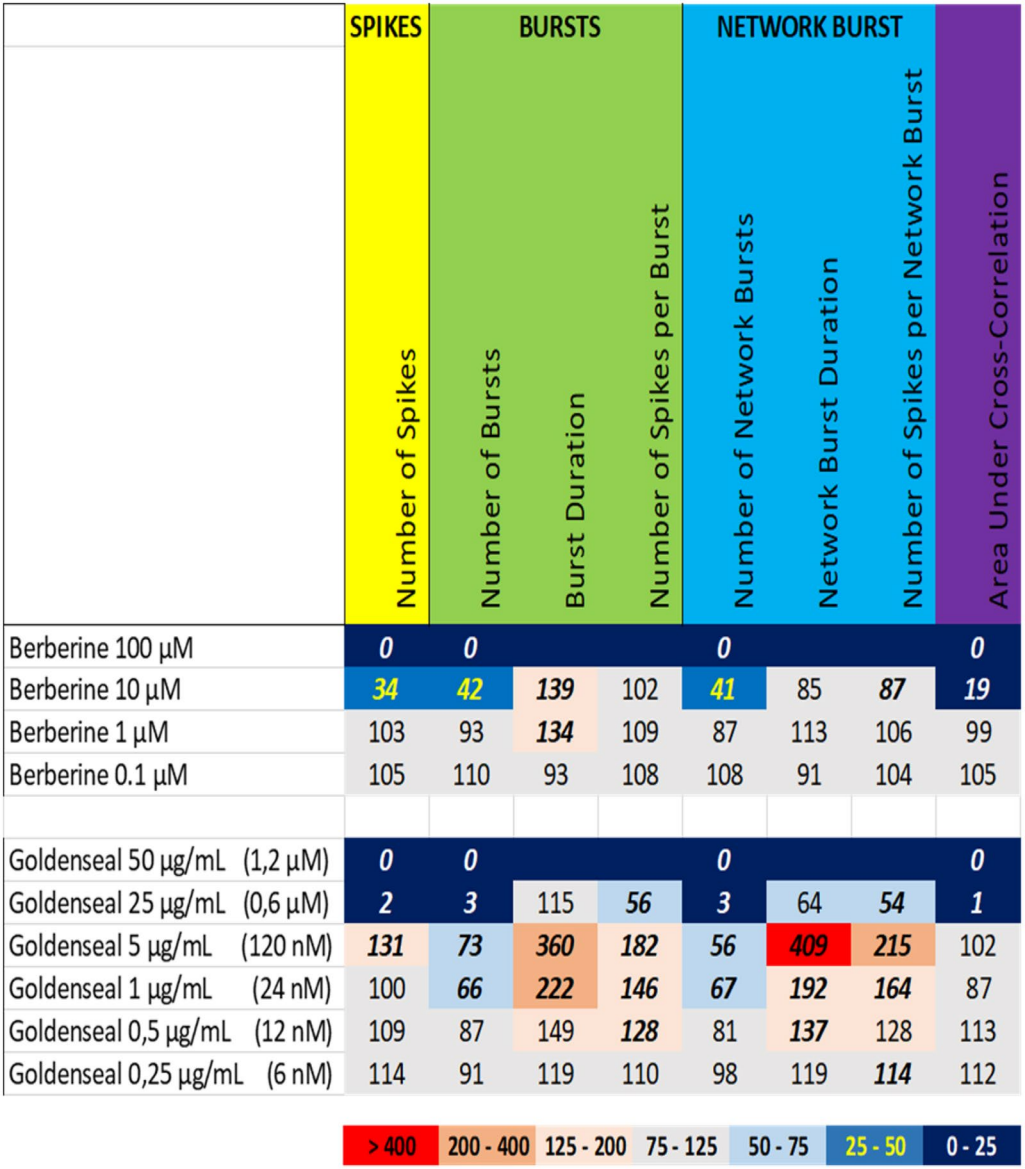
Overview of the effects of berberine (top) compared to the extract of goldenseal (bottom) on eight neuronal activity parameters. Effects are expressed as a percentage of DMSO control and the degree of effect is indicated using a color scheme. Decreases are depicted in blue, increases are depicted in red. Values that do not exceed the benchmark response (BMR) of 25%, which is derived from the variation of DMSO controls, are depicted in light grey and are considered to be of limited toxicological relevance. Values in bold deviate significantly from DMSO control (*p* < 0.05). The concentration of berberine in the goldenseal extract is indicated in between parentheses.

Exposure to the extract of goldenseal at 25 µg/mL and 50 µg/mL (equivalent to 0.6 µM and 1.2 µM berberine, respectively) resulted in a (near) complete inhibition of neuronal activity (Figure 13 (bottom), Supplemental Figure S12), whereas goldenseal extract at 1 µg/mL and 5 µg/mL (equivalent to 0.02 µM and 0.12 µM berberine, respectively) evoked a clear increase in the (network) burst duration and the number of spikes per (network) burst. This increase was not observed following exposure to berberine at 0.1 µM (Figure 13 (top), Supplemental Figure S20), suggesting that the goldenseal extract contains additional bioactive constituents with a specific activity phenotype. On the other hand, exposure to berberine at 10–100 µM resulted in a near complete cessation of neuronal activity as also observed following exposure to the high dose of goldenseal extract. However, the extract is far more potent in inhibiting neuronal activity than berberine alone.

**Figure 14.**
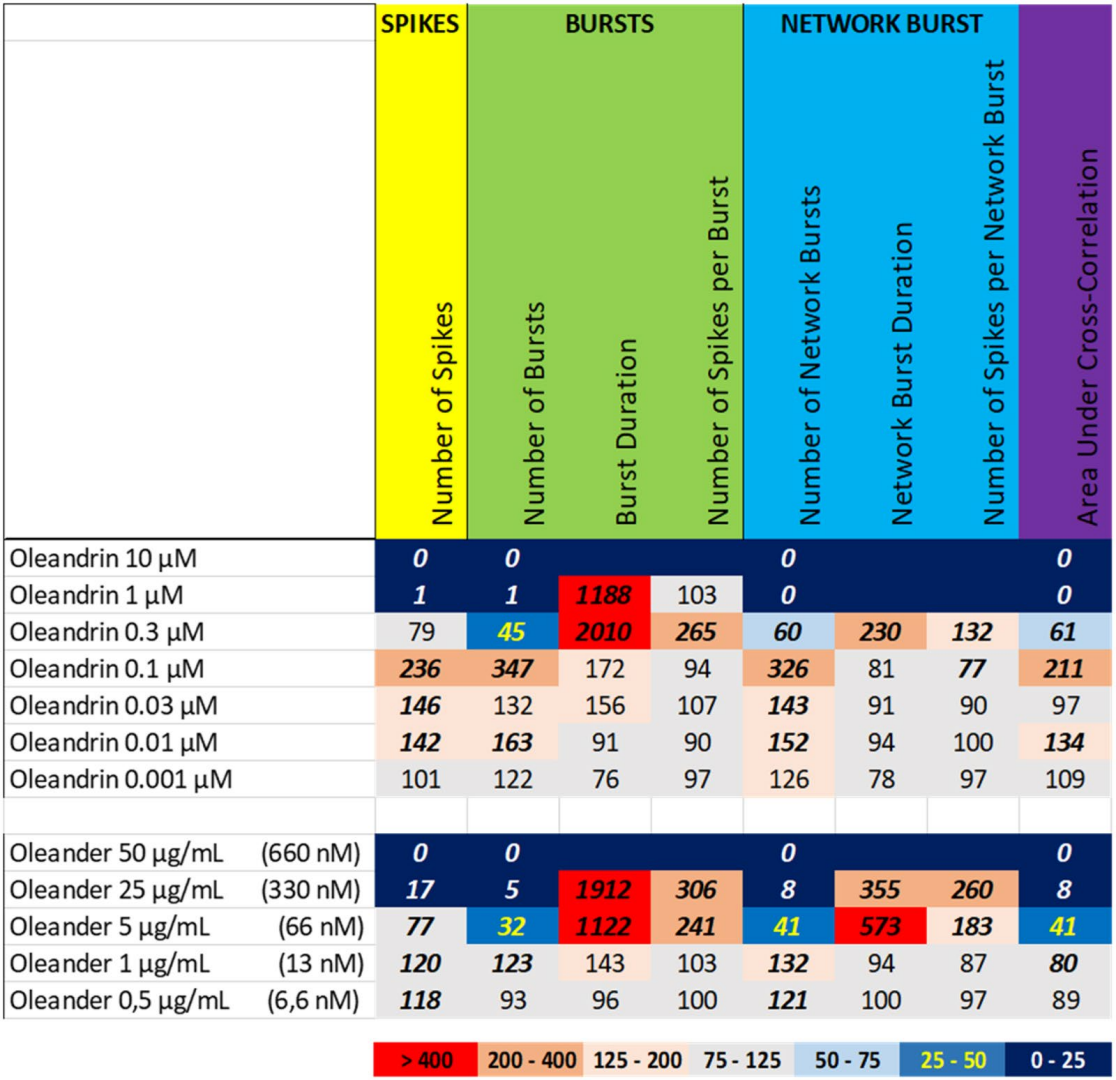
Overview of the effects of oleandrin (top) compared to the extract of oleander (bottom) on eight neuronal activity parameters. Effects are expressed as a percentage of DMSO control and the degree of effect is indicated using a color scheme. Decreases are depicted in blue, increases are depicted in red. Values that do not exceed the benchmark response (BMR) of 25%, which is derived from the variation of DMSO controls, are depicted in light grey and are considered to be of limited toxicological relevance. Values in bold deviate significantly from DMSO control (*p* < 0.05). The concentration of oleandrin in the oleander extract is indicated in between parentheses.

Exposure to the extract of oleander at 50 µg/mL (equivalent to 0.66 µM oleandrin) resulted in a complete inhibition of neuronal activity (Figure 14 (bottom), Supplemental Figure S13), whereas oleander extract at 5 µg/mL and 25 µg/mL (equivalent to 0.07 µM and 0.33 µM oleandrin, respectively) evoked a strong increase in the (network) burst duration and the number of spikes per (network) burst. This activity phenotype was only partially resembled by oleandrin, which at a concentration of ≥1 µM resulted in a (near) complete inhibition of neuronal activity (Figure 14 (top), Supplemental Figure S21), indicating that the inhibitory potency of the extract is somewhat stronger than oleandrin alone. The increased burst duration observed following exposure to 1 µM oleandrin may not be very reliable given the low number of bursts. However, oleandrin at 0.1 µM also induced a strong increase in (network) burst duration and the number of spikes per (network) burst, just as is observed for oleander extract. Oleandrin at 0.1 µM, 0.03 µM, and 0.01 µM evoked an excitatory effect with an increased number of spikes and (network) bursts, which was different from the burst intensification observed following exposure to the oleander extract at 5 µg/mL and 25 µg/mL.

## Discussion

Some of the botanicals selected for this study had or still have ethnomedical or traditional indications, including neurological. For example, blue cohosh is used by midwives to help induce labor, goldenseal is marketed in dietary supplements for the cold and flu, allergies, and gastrointestinal complaints, kratom is used to alleviate pain, kava has purported anti-depressant and anxiolytic effects, oleander has been used in ethnomedicine to treat asthma, eczema, ringworm, congestive heart failure, indigestion, and neurodegenerative diseases. In Africa, yohimbe is used to treat fever, leprosy, cough, and erectile dysfunction. Moreover, in Chinese medicine ephedra has been used to treat various conditions, such as colds, fever, headache, whereas tripterygium has been used to treat a variety of inflammatory and autoimmune disorders (reviewed in Kanungo et al. 2024). Despite these potential therapeutic benefits, we earlier showed the neuroactive potential of selected botanical extracts using a screening approach in *in vitro* rodent cortical cultures (van Kleef et al. 2024). With the current study, we extended the tested dose range to gain further insight in the neuronal activity phenotypes, to allow for effect categorization, and to derive dose-response relationships.

We previously demonstrated that exposure to extracts from ashwagandha (*Withania somnifera* (L.) Dunal, Solanaceae), comfrey (*Symphytum officinale* L., Boraginaceae), and Asian ginseng (*Panax ginseng* C.A. Mey., Araliaceae) evoked little change in neuronal activity (van Kleef et al. 2024). We therefore did not perform further dose-response testing for these three botanical extracts. The dose-response data are in good agreement with the earlier screening study (van Kleef et al. 2024). At 50 μg/mL, the extract of milk thistle induced a mild decrease in mitochondrial activity (9%; van Kleef et al. 2024), suggesting that the observed inhibition of neuronal activity at this dose could to a minor extent be due to a mild decreases in cell viability.

For the tested botanicals, extracts from milk thistle, usnea, and green tea produce a modest inhibition that affects primarily the number of spikes and (network) bursts (Figure 1). In contrast to this ‘simple’ inhibition, extracts from aristolochia and ephedra not only reduce the number of spikes and (network) bursts but also evoke a minor increase in (network) burst duration and/or number of spikes per (network) burst, i.e. a mild intensification of bursting (Figure 2). The different inhibitory phenotypes suggest that these extracts exert distinct and/or multiple modes of action. Tripterygium and yohimbe extract reduce neuronal activity more potently but also elicit a mild prolongation of (network) bursting in case of tripterygium (Figure 3). On the other hand, kava and kratom extract evoke a ‘simple’ inhibition, with a complete cessation of activity at the highest dose levels (Figure 4), although kava extract drastically increases burst duration in a narrow dose range. The distinct excitatory effects of aconite, blue cohosh, goldenseal, and oleander extracts further highlight the existence of multiple modes of action (Figure 5). Moreover, also within an activity phenotype multiple modes of action may exist as evidenced by for example the clear biphasic dose-response relationships with full inhibition at the high dose and extensive hyperexcitation at lower dose levels as observed for goldenseal and oleander extracts. Unfortunately, however, elucidation of the exact mode(s) of action requires the use of an extensive battery approach in which individual mechanisms, like inhibition/activation of distinct ion channels or (ant)agonism of specific neurotransmitter receptors, can be interrogated.

The obtained dose-response relationships not only aid in categorization of the extracts, but also allow for deriving No Observed Effect Levels (NOELs). As is evident from Table 1, only a few extracts (4/13) show neuromodulatory effects at levels below 1 µg/mL. Even extracts like those from kratom that evoke a complete cessation of activity at the highest dose, have a NOEL at 1 µg/mL. Only for yohimbe, kava and oleander extracts the NOELs amount to 0,5 µg/mL or 0,25 µg/mL in case of goldenseal, indicating a relatively narrow effective range for the various botanical extracts.

**Table 1.** Overview of the No observed effect levels (NOELs) on the eight neuronal activity parameters following exposure to the 13 different botanical extracts.

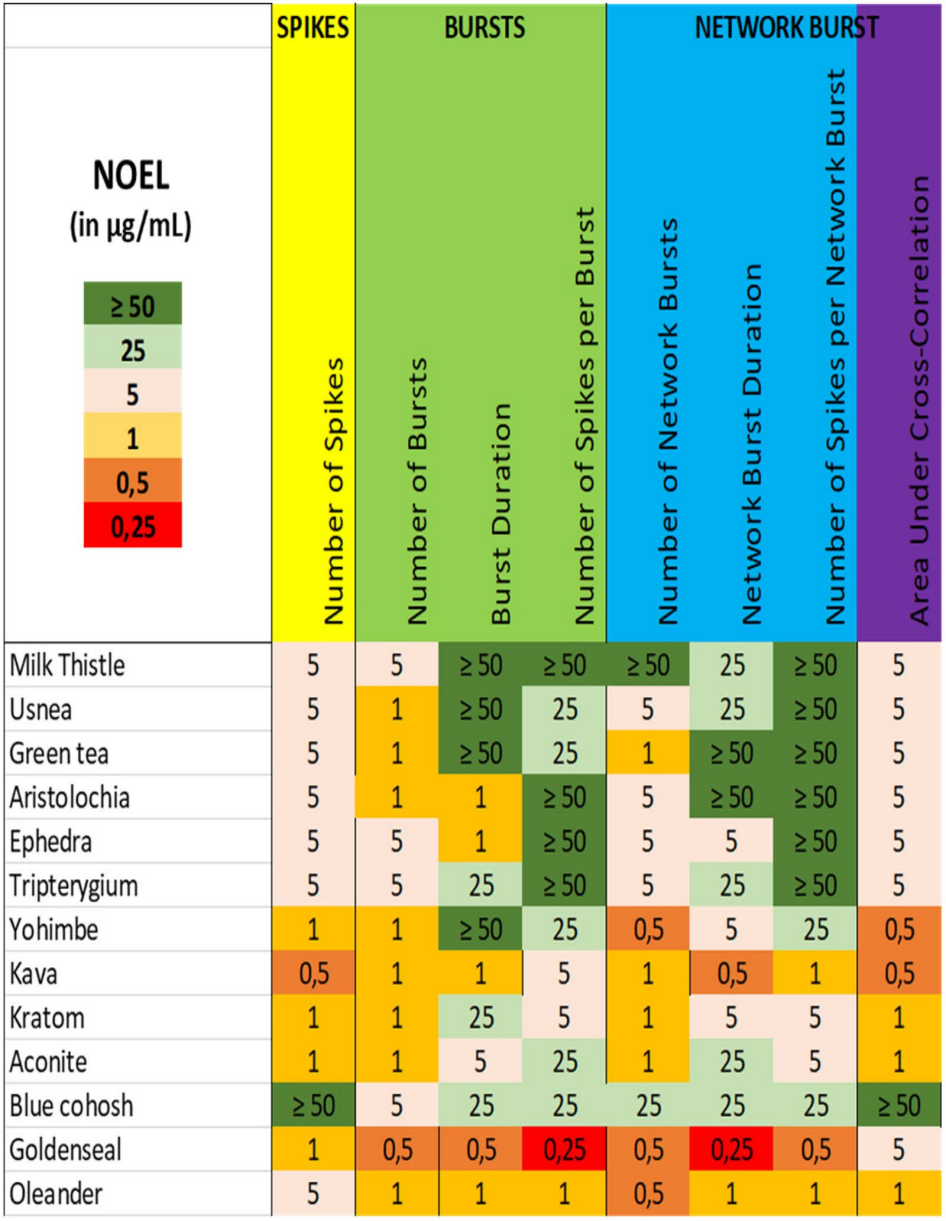

NOELs are expressed in µg/mL and color-coded with values in red being most potent and values in green least potent.

From Table 1 it can also be concluded that the number of spikes and/or the number of (network) bursts are generally among the most sensitive parameters. Goldenseal extract is an exception where (network) burst duration and the number of spikes per (network) burst are the most sensitive parameter. Oleander extract is another exception as it shows very little difference in the NOEL for the different activity parameters. This diversity in NOELs, combined with the biphasic effects observed for the extracts of oleander, aconite, and goldenseal, again points toward distinct modes of action as well as distinct bioactive constituents. As already mentioned above, identification of the exact mode(s) of action will be a challenging and laborious task, but identification of the bioactive constituent(s) in the extracts may ease identification of the modes of action.

We therefore also tested a number of individual constituents from the extracts for their effects on neuronal activity (see Supplemental Table S2 for concentrations of constituents in the extracts and how the tested concentrations of constituents relate to the different tested dose levels of the extracts). The tested extracts generally exert an effect that is larger or occurs at a lower dose compared to the constituents (at equimolar levels), indicating the higher potency of the extract. The constituents silybin B and epigallocatechin gallate do not evoke an activity phenotype that is comparable to the extracts of milk thistle and green tea, respectively (Figures 6 and 7). This clearly indicates the presence of additional neuroactive constituents in the extracts. Dihydrokavain, berberine and oleandrin show an activity phenotype that partially resembles the neuroactive effects of the extracts of respectively kava, goldenseal and oleander (Figures 10, 13, and 14), also pointing toward the presence of additional neuroactive constituents in the extracts. Notably, yohimbine, mitragynine and aconitine do evoke an activity phenotype that is comparable to the extracts of respectively yohimbe, kratom and aconite (Figures 9, 11, and 12). While this may suggest that yohimbine, mitragynine and aconitine are indeed the bioactive constituents responsible for the observed neuroactive effects, it should be noted that the extracts are more potent than the constituents (at equimolar levels) alone and the extracts thus likely contain additional neuroactive constituents that act additively or even synergistically with the tested constituents. These findings indicate that identification of the bioactive constituent(s) in the extracts requires a laborious approach in which more constituents must be isolated and tested individually and as a mixture, further highlighting the complexity of hazard characterization of botanical extracts.

Deriving NOELs of the botanical extracts and their constituents is an important first step in the attempt to relate these *in vitro* findings to human exposure levels and possible health effects. However, this effort is strongly hampered by the scarcity of *in vivo* and human data which precludes for example *in vitro*-*in vivo* extrapolation (IVIVE). While there are many reports on human health effects of botanical extracts, the corresponding intake levels are often poorly specified and analytical characterization of the extract is lacking. Similarly, there is little information in literature regarding human blood or plasma levels of bioactive constituents following intake of the tested botanical extracts.

For the eight tested constituents in our study, only for yohimbine, mitragynine, aconitine, and oleandrin scarce case study reports provide human blood levels. For yohimbe, case reports provide fatal yohimbine blood levels ranging from 5.4 to 7.4 μg/mL (corresponding to 15 to 21 μM; Anderson et al. 2013), which is close to the concentrations of yohimbine that exert a clear inhibition of neuronal activity (1–10 μM; Figure 9). For kratom, case reports provide postmortem mitragynine blood levels ranging from 0.016 to 4.8 μg/mL (corresponding to 0.040 to 12 μM), with the majority of cases showing blood levels below or close to 1 μM (Kronstrand et al. 2011; Eggleston et al. 2019; Gershman et al. 2019). These (fatal) blood levels are close to the concentrations of mitragynine that exert a clear inhibition of neuronal activity in our study (Figure 11). For aconite, (survival) case reports provide aconitine blood levels ranging from 0.2 to 2 ng/mL (corresponding to 0.3 to 3 nM; Moritz et al. 2005; Niinuma et al. 2002), which is close to the concentrations of aconitine that exert a clear hyperexcitation (10–100 nM; Figure 12). Finally, for oleander, case reports provide fatal oleandrin blood levels ranging from 10 to 37.5 ng/mL (corresponding to 17 to 65 nM; Arao et al. 2002; Carfora et al. 2021; Okuda et al. 2024; Wasfi et al. 2008), which is close to the concentrations of oleandrin that exert a clear hyperexcitation (100 nM; Figure 14). While these scarcely reported (fatal) blood levels are often close to the effect levels obtained in our *in vitro* study, levels in the brain can be very different e.g. due to poor blood-brain barrier translocation. It is thus important to point out that human blood levels cannot be one-on-one compared to the effect levels reported here. Future research on toxicokinetic and human exposure levels is therefore essential to causally relate our *in vitro* findings to possible human health effects.

## Conclusions

Exposure to botanical extracts and constituents affects neuronal network activity *in vitro*, resulting in specific activity phenotypes with a narrow effective dose range. Different inhibitory phenotypes as well as distinct excitatory phenotypes are observed, indicative for multiple modes of action. Most of the tested constituents resemble the effect of the respective botanical extracts. The extracts generally have a higher potency than the individual constituents, indicative for the presence of additional neuroactive constituents that act additively or even synergistically with the tested constituents, further highlighting the complexity of hazard characterization of botanical extracts.

## Supplementary Material

HESI dose_resp supplemental data.docx

Supplemental_Chemical_Analysis_Tripterygium.pdf

Supplemental_Botanical_Constituents_and_Quantification_PB.xlsx

## Data Availability

The data that support the findings of this study are available from the corresponding author, RHS Westerink, upon reasonable request.
